# Deep subwavelength ultrasonic imaging using optimized holey structured metamaterials

**DOI:** 10.1038/s41598-017-08036-4

**Published:** 2017-08-10

**Authors:** Kiran Kumar Amireddy, Krishnan Balasubramaniam, Prabhu Rajagopal

**Affiliations:** Centre for Nondestructive Evaluation and Department of Mechanical Engineering, Indian Institute of Technology Madras, Chennai, 600036 Tamil Nadu India

## Abstract

This paper reports the experimental demonstration of deep subwavelength ultrasonic imaging of defects in metallic samples with a feature size of λ/25 using holey-structured metamaterial lenses. Optimal dimensions of the metamaterial’s geometric parameters are determined using numerical simulation and the physics of wave propagation through holey lenses. The paper also shows how the extraordinary transmission capacity of holey structured metamaterials comes about by the coupling of higher frequencies in the incident ultrasonic wave field to resonant modes of the lens.

## Introduction

Ultrasound is widely used as a modality for diagnostics in measurements and imaging for engineering materials, components and processes as well as in biomedicine, owing to its affordability, absence of radiation risk and ability to probe deeper into thicker samples. However, conventional methods in ultrasound suffer from poor resolution, (limited by classical Rayleigh scattering limit^1^ to λ/2 where λ is the operating wavelength) typically  in the range of millimeters. Most Non-Invasive Diagnostics (NID) (recently even Non-Destructive Evaluation (NDE)) applications such as imaging of tissue and bone on the other hand, demand resolution in the low micrometer to nanometer range. Ultrasonic excitation in the order of GHz can potentially yield such resolution, but this leads to very poor penetration into the structure. Thus expensive and radiation-prone electromagnetic (EM) wave approaches (e.g. X-ray) are often the only solution, increasing the cost of evaluations (or treatment in biomedicine). Sometimes such high resolution evaluation and diagnostics is just not possible, for example when sample thickness is in the order of centimeters.

Several strategies to improve the resolution^1^ of lower frequency (with deeper material penetration) ultrasonic evaluation have been considered in literature^2–5^, but typically the methods are limited to post-processing of raw imaging data, and removal of noise becomes a major challenge in such approaches. In this context, research by the authors has sought to enhance the resolution of ultrasonic evaluations using holey-structured metamaterials. Emerging from extensive research worldwide over the last decade in the context of EM and acoustic waves, metamaterials typically consist of artificially engineered structures with special properties that are otherwise not found in nature^6–17^.

Among sonic metamaterial concepts (see^18^, ^19^ and references therein for example), holey structures^14^ are relatively easier to create and also to manipulate to achieve specific properties such as extraordinary transmission or absorption. Using holey structured lenses, the authors recently demonstrated experimentally, a subwavelength resolution of λ/5 for the first time in the ultrasonic regime (see ref. 20). In the holey structured metamaterial lens each ‘hole’ acts as a pixel for imaging and improves the resolution by amplifying the decaying evanescent waves through Fabry-Perot resonant modes^18^. Resonance in turn depends on the geometrical parameters of the metamaterial (diameter, length and periodicity of the holes) and frequency of excitation as explained for example, in^20^. At the Fabry-Perot resonance condition, the transmission coefficient approaches unity and hence the meta-lens transfers all the waves (including evanescent waves) from object plane (input surface) to image plane (output surface) without any loss. The typically high frequency evanescent wave components contain subwavelength information, yielding super-resolution.

The present paper builds on this previous work^20^, and shows that optimization of the lens structural parameters can enable much deeper subwavelength resolution. We present a detailed study of the physics underlying the performance of holey-structured metamaterial lenses. We also discuss optimal geometrical parameters (namely, hole size, length and period) to achieve extraordinary transmission. Finally, with the help of a metalens fabricated according to the optimal parameters, we experimentally demonstrate a deep subwavelength resolution of λ/25 in the ultrasonic regime. To our knowledge, this is the best experimental resolution demonstrated for defect characterization in the ultrasonic regime. We also present an experimentally-generated deep subwavelength ultrasonic image of a λ/25-long through-notch in a metallic sample, perhaps the first such demonstration worldwide.

## Results

Firstly we discuss studies on the optimization of geometric parameters of the holey structured metamaterial lens. An optimized metalens is then used to experimentally demonstrate a deep subwavelength resolution of λ/25 for ultrasonic line scan (B-scan) of a metal block with side-drilled circular holes (a type of defect feature commonly used to study imaging resolution). Finally, the optimized metalens is also used to obtain line and 2D (C-scan, see R﻿ef. 22) images of a metal block with a narrow-width through-notch/crack of deep subwavelength dimension of the order of λ/25 (as an example of a typical defect type encountered in NDE applications).

### Optimal geometrical parameters of the metalens

**Figure 1:**
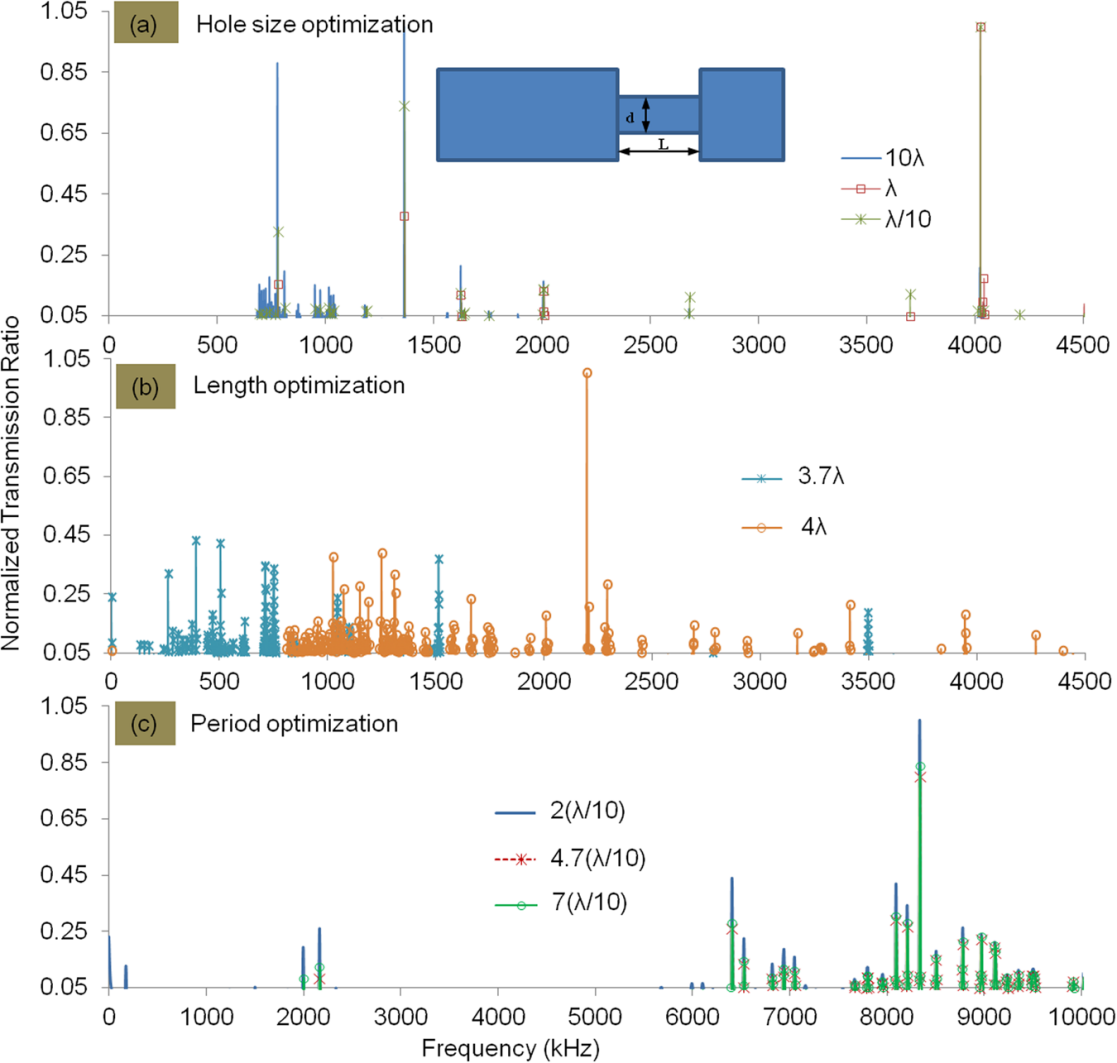
FE results for Transmission Ratio (TR) of metamaterial with varying geometrical parameters of metamaterial lens, for different values of (**a**) Hole size (**b**) Hole length and (**c**) Periodicity (To avoid crowding of data points, for all above graphs the starting value on the ordinate is considered as 0.05 instead of 0). A full set of results is presented in Supplementary Figure 3 in Additional Information.

Optimal geometrical parameters of the metalens were first studied with the help of Finite Element (FE) simulations. This exercise is necessitated by the fact that although information on optimal ranges for some of the individual geometrical features is known from literature, a unified guideline for all parameters simultaneously is not available. The modelling approach is the same as that used in our earlier study^20^ and well-validated with experiments presented therein. For better readability, only a subset of results from these studies is presented in Fig. 1 in the main body of the paper, while a full set of results is given under ‘Additional Information’ (see Supplementary Figure 3).

A very important and critical parameter of the periodic holey-structured metamaterial is the hole size (diameter). For this set of optimization studies, we considered models with a single (unit) hole (see inset image in Fig. 1(a)). The Transmission Ratio (TR, or spectral ratio of signals transmitted with and without the metamaterial, see ref. 20) of the lens (was calculated for different cases where the hole diameter was varied with values: large (10λ), small (λ/10) and comparable to the wavelength (*λ)* while keeping the cell length constant. This set of studies showed that a hole size (λ/10), resulted in high TR as shown in Fig. 1(a). We observed that a very small hole size (λ/20) also has similar TR values as that of the λ/10 case (See Supplementary Figure 3(a) in Additional Information for full set of results) and hence the hole size is taken as λ/10 for all further studies.

Next, the TR was calculated maintaining the hole size as λ/10, while the cell length was varied from *λ/2* to *15λ* (to satisfy the Fabry-Perot resonance condition, the cell length should be an integer multiple of half the operating wavelength^21^). As shown in Fig. 1(b), we observe that for hole length taking values that are integer multiples of half wavelengths, (See Supplementary Figure 3(b) in Additional Information for full set of results for different length cases including integer and fractional multiples of half wavelength) the TR is good. However, for cases where the hole length takes values that are fractional multiples of the half wavelength, the TR is low. We selected a hole length value of *4λ* where the TR has a clear peak at high frequencies, for further studies.

For further optimization studies, a full model with many holes was used (see ref. 20 for details of FE implementation). In these models, the TR was calculated with optimal hole size and length taken to be λ/10 and 4λ respectively, while the hole-hole distance (periodicity) was varied from 2(λ/10) to 7(λ/10). Corresponding results (to avoid crowding of data points only one case representing even, odd and fractional multiples of optimum hole length (λ/10) are shown in Fig. 1(c). Please see Supplementary Figure 3(c) in Additional information for further results) are shown in Fig. 1(c). We find that the TR is high for periodicity taking even multiple values of λ/10 (optimum hole diameter), where the transmitted resonance wave field from all holes interfere constructively.

We can summarize results of the above studies on optimal parameters of the holey structured metamaterial lens as follows. For better resolution, diameter of the holes should be *λ/n*, where *n* is an integer of value >  = 10. The hole length should be an integer multiple of half the wavelength *m*(*λ/2*), as expected based on the Fabry-Perot resonance principle^20^. The hole periodicity should be *(2p)(λ/n)*, where *p* is an integer (See Additional Information for discussion on this point).

Further if *w* is the wall thickness between the two holes, the periodicity can be written as the sum of the hole size and the wall thickness, thus the optimal wall thickness can be shown to be (See Additional Information)

1 $$w=(2p-1)(\frac{\lambda }{n})$$

### Applications of the metamaterial lens for imaging of subwavelength features in the materials

**Figure 2:**
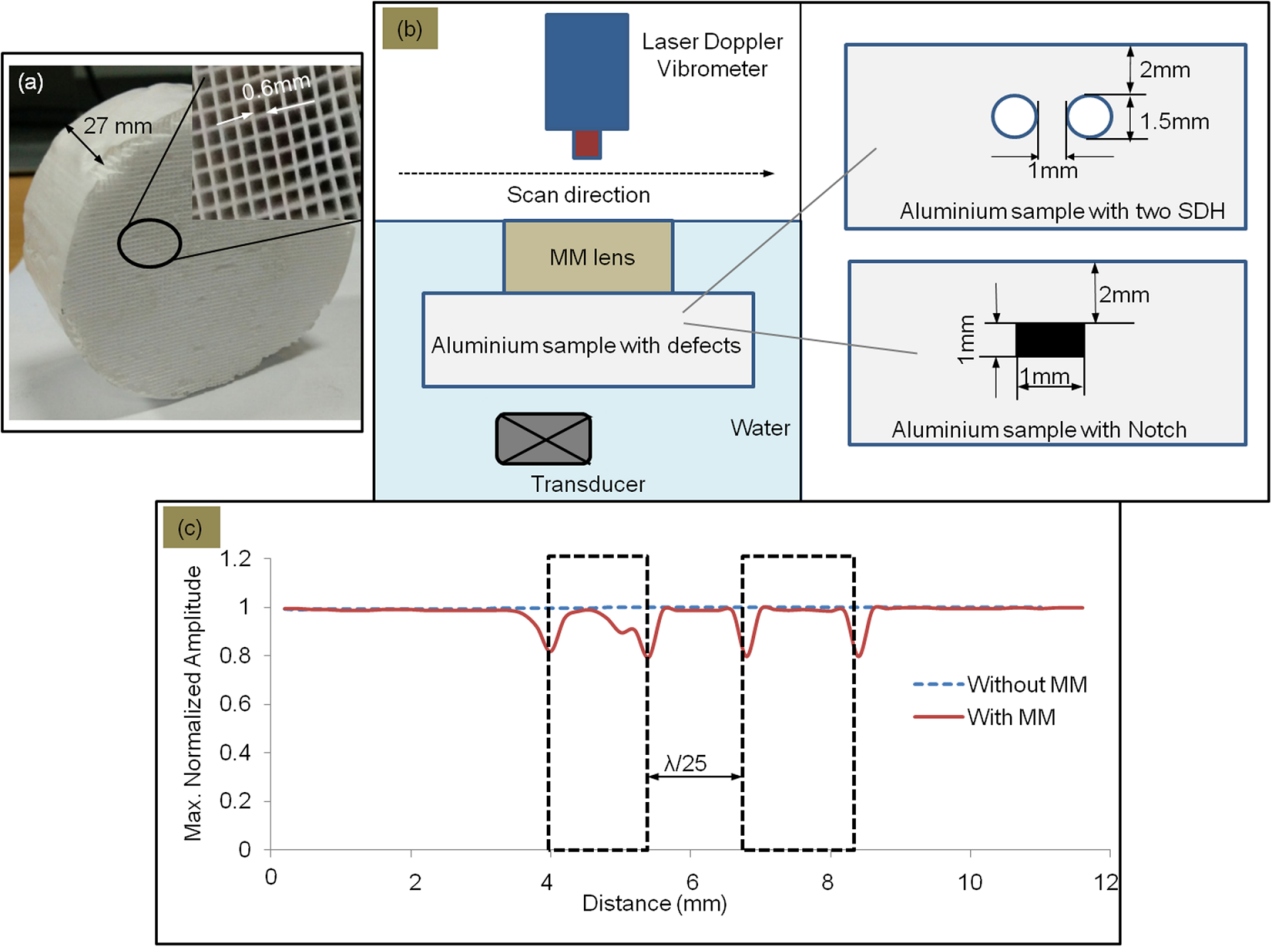
(**a**) Photograph of the holey structured metamaterial lens made with optimal parameters; (**b**) Illustration of experimental set-up (In-set details of two types of defects considered in an aluminium sample); (**c**) Experimental results for maximum normalized amplitude variation with the monitored positions across the sample: the two dotted rectangular boxes indicate actual positions of the defects in the aluminium sample.

We fabricated a holey structured lens with the optimal geometrical parameters as per above considerations to experimentally demonstrate deep subwavelength resolution and imaging using ultrasound interrogation. The details of the lens are as follows: the hole size *d* is 0.6 mm (λ/10 for longitudinal waves in water at 244 kHz), length *L* is about 27 mm (which is 9(λ/2) for longitudinal waves in water at 244 kHz) and the period *Λ* is 1.2 mm (about two times the hole size) as shown in the Fig. 2(a).

### Deep subwavelength resolution of side-drilled holes separated by λ/25

Firstly in order to demonstrate resolution in the deep subwavelength regime, as with the earlier study^20^, we considered a metal block with Side Drilled Holes (SDH) of subwavelength size: the SDH were each of 1.5 mm diameter separated by 1 mm (λ/25 at a central frequency of 244 kHz in the aluminium sample for longitudinal ultrasonic wavelength). The SDH were located at a depth of 2 mm (λ/18) from surface of the sample. As shown in Fig. 2(b), this defective sample is considered as the object for imaging using ultrasonic immersion scan in through-transmission mode. A line scan (B-scan) image was obtained without and with the metamaterial lens. The corresponding results are shown in Fig. 2(c). Without the metalens, the subwavelength sized holes in the aluminium sample act only as perturbations (detailed explanation is given in Discussion section below) and cannot be resolved in the image. However the use of metalens made of optimum geometrical parameters, leads to the deep subwavelength resolution of λ/25, far below the diffraction limit.

### Deep Subwavelength image of a through-notch of size λ/25

To demonstrate ultrasonic imaging in the deep subwavelength range, we considered a metallic block with a side-drilled notch. A subwavelength through-notch of length 1 mm (which is about λ/25) and located 2 mm (λ/18) from the surface in an aluminium sample as shown in Fig. 2(b) is considered as the object for ultrasonic imaging.

**Figure 3:**
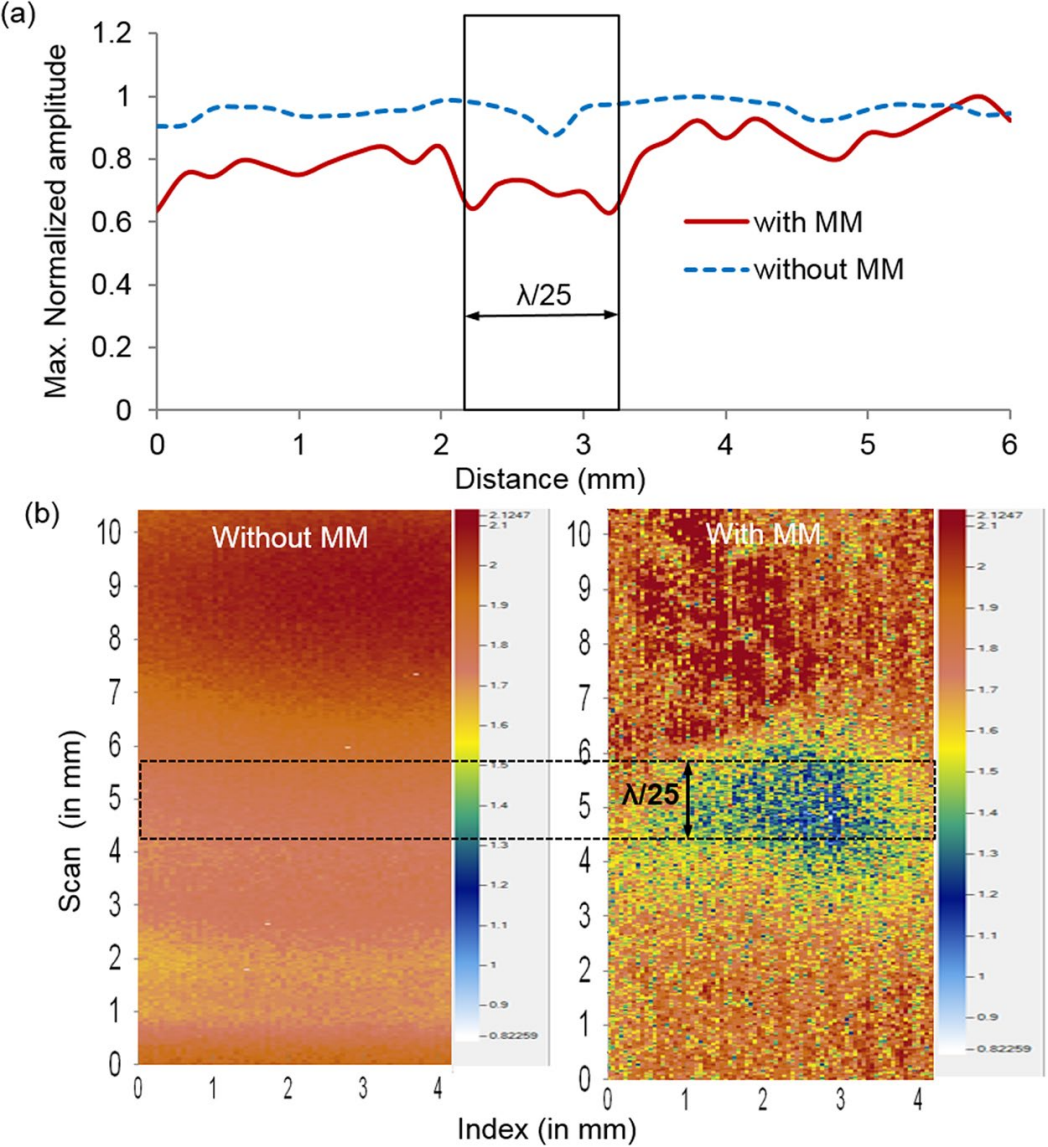
(**a**) Experimental results for maximum normalized amplitude variation with the monitored positions across the sample: the rectangular box indicates the actual positions of the notch in the aluminium sample; (**b**) Experimental results for ultrasonic C-scan image of through-notch in the aluminium sample without and with metamaterial lens: the dotted rectangular box indicates the actual positions of the notch in the aluminium sample.

The B-scan obtained (using the same procedure as for the resolution study described above) without and with metamaterial lens is shown in Fig. 3(a). At the notch position (indicated by the black coloured rectangular box), even without the metamaterial, a drop in the signal amplitude can be observed, but we are unable to resolve the dimensions. However when the metamaterial mediates the signals received by the Laser vibrometer pickup, there is a clear drop in the signal amplitude at the defect location positions. This is because the backscatter component, occurring at higher frequencies (see ‘Discussion’ section for more details on the physics of this phenomenon), is not recovered when waves are transmitted past the metalens. Similarly, diffraction from the edges of the notch can also be well-differentiated.

While the line (B-) scan above gives an indication of the defect, a 2D image is often necessary to obtain a more detailed understanding. Towards this end, a C-scan^22^ of the sample was also performed in immersion ultrasonic through-transmission mode. The transmitter probe was excited at a central frequency of 244 kHz, while the scattered wave field was received by a Laser Doppler Vibrometer. The experiment was performed without and with the metalens, and corresponding C-scan imaging results are shown in Fig. 3(b). We observe that the subwavelength (λ/25) notch shows up well when using the metalens. This is perhaps the first demonstration of deep subwavelength imaging in the ultrasonic regime worldwide.

## Discussion

**Figure 4:**
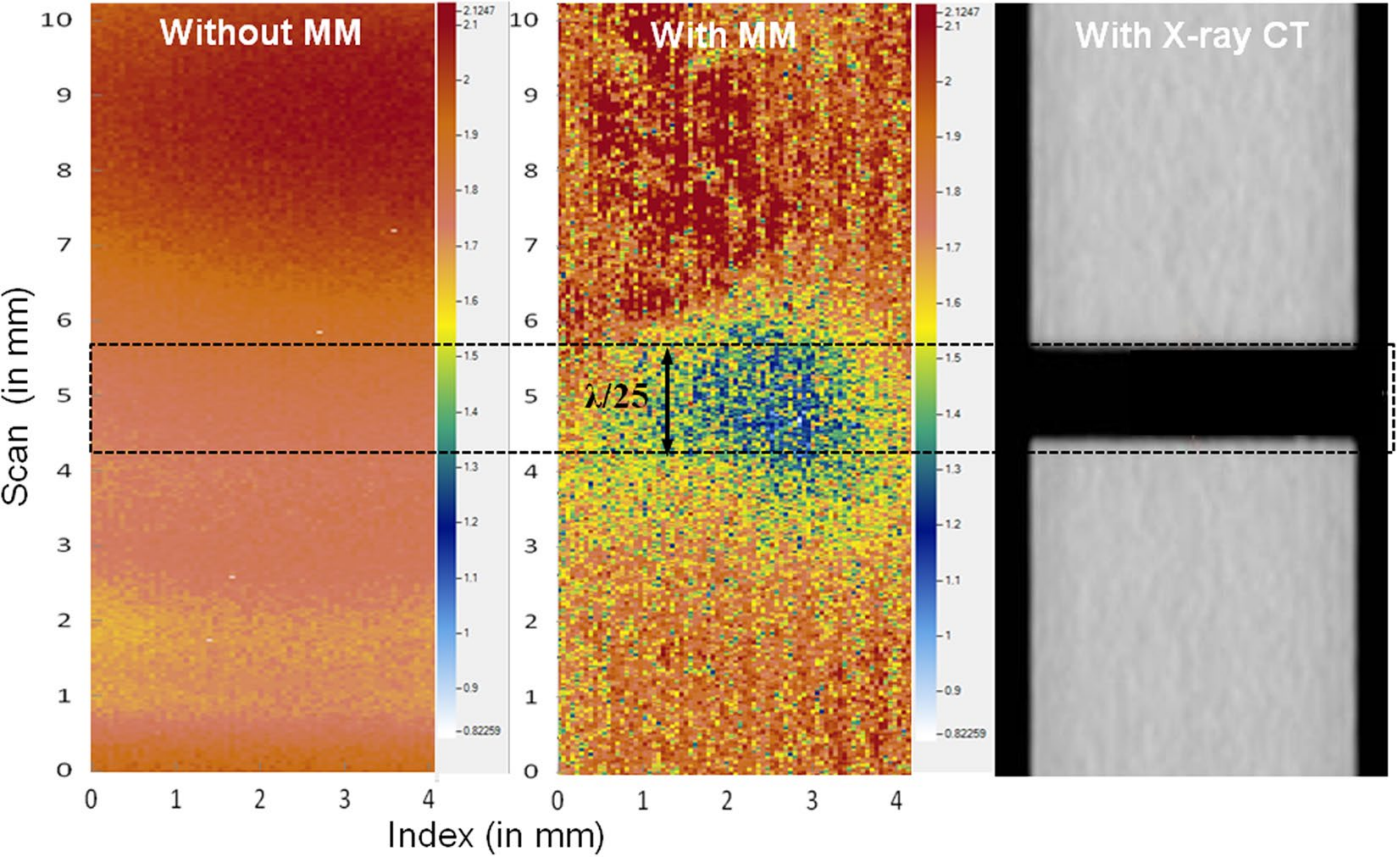
Image obtained by ultrasonic immersion C-scan without and with metamaterial lens, in comparison with that from X-ray μCT scan.

The image obtained by ultrasonic immersion C-scan mediated by the metamaterial lens was compared with that from micro X-ray Computed Tomography (µCT)^22^ as shown in Fig. 4. The crack length observed in the μCT- scan is about 1.02 mm, whereas the mean Full width Half Maximum (FWHM, see ref. 23) taken from the ultrasonic image (C-scan amplitude data) is about 1.061 mm, which agrees well with the μCT value. This clearly demonstrates the resolving capacity of the metamaterial ultrasonic superlens developed here. The working principle or physics behind the resolving capacity of this lens is discussed below.

**Figure 5:**
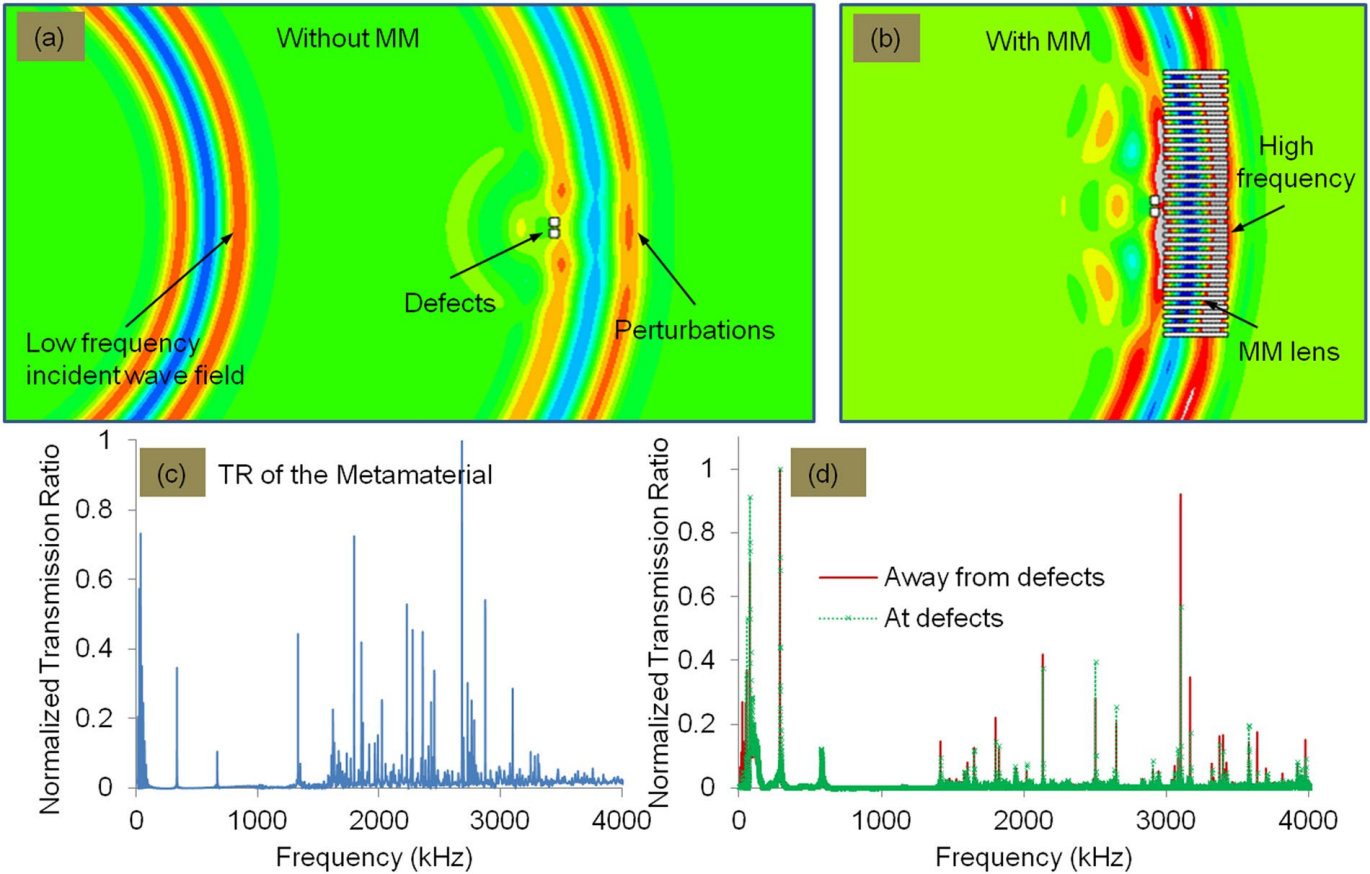
Illustration of wave scattering at defects (**a**) Without and (**b**) With metamaterial lens (**c**) Experimental results showing typical TR of the metamaterial lens (**d**) TR of the metamaterial lens at and away from defect positions. At the defect position several frequency components are suppressed.

When low frequency waves (with larger wavelength) interact with a subwavelength feature, the feature acts as a perturbation. In this case as illustrated in Fig. 5(a), subwavelength features are not identified in the image due to the loss of the high frequency components (evanescent waves). Generally, scattering takes place at slightly higher frequency compared to incident waves and the effect would be seen in the close near-field only. When a wave field is transmitted through periodically perforated metamaterials, three concurrent physical phenomena happen, namely the Fabry-Perot resonance inside the holes, coherent scattering due to periodic hole array structure and elastic surface-modes (see for example, ref. 24). At the Fabry-Perot resonance, these scattered waves are coupled into the holes and get amplified while travelling through them. In addition, the effects of periodicity and size of the holes come together to yield coherent scattering^21^.

Holey-structured metamaterials act as filters for the scattered wave field and can be thought to selectively pass high frequency components from the input-to the output-surface (image plane). This can be thought to arise from the fact that wave components at higher frequencies, with wavelengths λ such that λ/2 < *d* (where d is the hole size), are more effectively transmitted past the holes of the metamaterial. Thus with holey metalenses, the high frequency components scattered by defective features get selectively amplified and are transferred to the image plane as illustrated in Fig. 5(b). This can be seen for example from the results in Fig. 5(c), showing the typical frequency content of the TR monitored in the experiments with the metalens (See Additional Information for TR variation with different input frequencies). Moreover, we also observe that some of the high frequency components are lost in the signals monitored at the defect region compared to the signals monitored away from the defect region, as shown in Fig. 5(d).

Some of these frequency details are also shown collected together for comparison in Table 1. We observe that at the position of defects, the low frequency components are preferentially amplified, while high frequency components are suppressed. This is expected, since higher frequencies would be more strongly scattered back at the position of the defects. In conventional imaging this detail is lost, as the low frequency components overpower the overall signal content. However, when we image using the metamaterial, we are essentially amplifying higher frequency components transmitted, and imaging at finer wavelengths. Hence, at the position of the defects where significant part of energy at higher frequencies is backscattered, the overall time-domain amplitude drops: thus the resolution improves greatly.

**Table 1:** Amplitude of Transmission Ratio (TR) at the defects and away from the defects at various frequencies.

Low frequency components		
Frequency (kHz)	Amplitude At defect	Amplitude Away from defect
55	0.531	0.089
76	0.913	0.530
287	0.724	0.710
575	0.124	0.106
High frequency components		
1413	0.041	0.144
1602	0.061	0.082
1799	0.146	0.222
2130	0.378	0.421
3098	0.570	0.922
3165	0.173	0.349
3396	0.116	0.165
3635	0.156	0.177
3974	0.094	0.153

It must be noted here, that all our calculations were performed assuming the speed of sound in the water-filled holes of the metamaterial to be the same as the speed of sound in unbounded water. In reality, each of the holes will act as a water-filled duct waveguide, and thus will support different guided wave modes (see for example, refs 25–27). For rigid circular ducts, the fundamental guided mode exists at zero frequency and its velocity is close to but slightly lower than sound velocity in the bulk of water, and fairly constant with frequency^25^. This mode also has particle motion closer to plane wave motion, and it can be assumed that a majority of incident wave energy is coupled to this mode in the duct. In our case, while dispersion curves have to be computed for a water-filled square duct and this is an ongoing effort at our group currently, we can assume safely that the wave energy is carried again primarily by the fundamental mode with a velocity close to that in the bulk of water. Thus our calculations based on sound speed (and wavelength) in water, work in an approximate sense.

Finally, while the defects considered in this paper are close to the surface, it is indeed possible to image features that are located deeper from the surface. The defective feature must be at a distance from the surface in the order of a maximum of one wavelength from the surface – this can be modified by changing the frequency of interrogation. We then have to choose a metamaterial lens with geometrical parameters suited to the resulting wavelength of interest (these parameter values can be calculated using the procedure presented here).

## Methods

The experiments were conducted in water immersion mode. A commercially available transducer of central frequency 180 kHz (Panametrics – GE Measurement and Control, Billerica, Massachusetts USA) is used as the source of ultrasound. The excitation, controlled by a RITEC 4000 Pulser-Receiver (Ritec Inc., Warwick, Rhode Island, USA), consisted of a 3 cycle Hanning windowed tone-burst signals driven at a central frequency of 244 kHz. Aluminium samples with (a) two side-drilled holes of diameter 1.5 mm and separated by 1 mm and (b) 1 mm long through-crack are placed in front of the transducer as considered as the objects for imaging. The holey-structured metamaterial is placed immediately in front of the aluminium sample such that, the scattered wave field from the object can be coupled into the metamaterial. The head of the OFV 551 Laser Doppler Vibrometer (Polytec GmbH, Waldbronn, Germany) is attached to a raster scanning system and used to measure the scattered wave field on the output surface of the metamaterial.

## Electronic supplementary material

Supplementary Information
