# Supplementary material for: Deep subwavelength ultrasonic imaging using optimized holey structured metamaterials
Source: Sci Rep. 2017 Aug 10;7:7777. doi: 10.1038/s41598-017-08036-4 (PMC5552794; doi:10.1038/s41598-017-08036-4)
Supplement: Supplementary file 1 — Supplementary Information [file 41598_2017_8036_MOESM1_ESM.pdf]

Deep subwavelength ultrasonic imaging using optimized holey structured metamaterials

Kiran Kumar Amireddy*, Krishnan Balasubramaniam and Prabhu Rajagopal

*Centre for Nondestructive Evaluation and Department of Mechanical Engineering, Indian Institute of Technology Madras, Chennai 600036,Tamil Nadu, India. (*Email: amireddykiran@gmail.com)*

Additional Information

Transmission ratio variation with different input frequencies

We used time-domain explicit finite element (FE) simulations implemented in a commercial package [1] to understand the frequency amplifying capacity of the holey-structured metamaterial for different input frequencies in the range of 100–700 kHz. (Details of the FE modeling approach and implementation can be found in our previous work [2]). For each input frequency case, the frequency at which maximal transmission occurs and the maximum high frequency up to which there is significant energy content in the transmitted signal were obtained, as shown in Supplementary Figure 1.These results help not only in understanding the frequency amplification capacity, but also to predict the maximum resolution achievable by a given metamaterial lens. This type of curve is perhaps characteristic of and can specify the performance of holey structured metamaterial lens with particular geometric parameters.

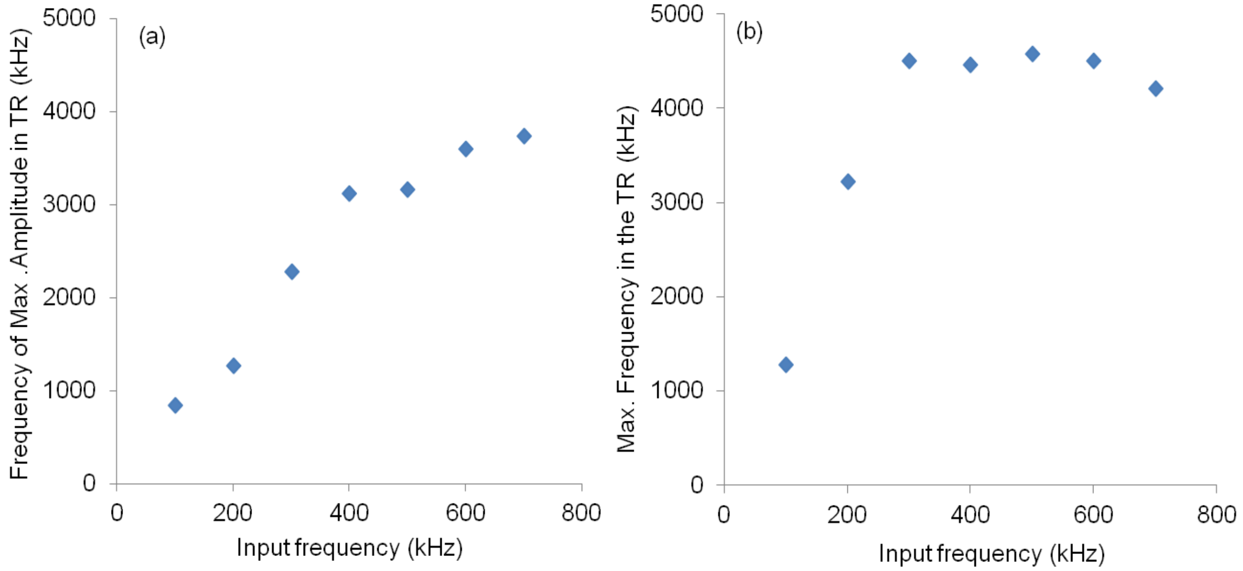

Supplementary Figure 1. FE simulation results showing (a) Variation of frequency at which transmission is maximal for various input frequency values, and (b) Maximum frequency up to which there is significant energy in the TR spectrum, with corresponding input frequency values.

Optimal geometrical parameters of the lens

We can summarize results of the studies on optimal parameters of the holey structured metamaterial lens as follows. For better resolution, diameter of the holes should be Λ/n, where n is an integer > =10.

Hole length should be an integer multiple of half the wavelength $m(\Lambda/2)$, as expected based on the Fabry-Perot resonance principle [2, 3].

The hole periodicity should be (2p)(Λ/n), where p is an integer. This can again be understood from the underlying physical principles of holey structured lenses [4]. Consider the expression for the Transmission Ratio (T) of the metamaterial lens (see [2, 4] for example):

$$T = \frac{4(\frac{d}{\Lambda})^2 Y\, e^{ikL}}{(1+Y\left(\frac{d}{\Lambda}\right)^2) - (1-Y(\frac{d}{\Lambda})^2)^2 e^{2ikL}} \qquad \text{----- (1)}$$

where, d is the hole size, Λ is periodicity, Y is the admittance of the waveguide mode within the holes, k is the wave number, and L is the length of the metalens.

Approximating the overall transmission as a sum of the all multiple scatterings in the holes, the above equation can also be rewritten as below:

$$T = \frac{\tau_{12}\tau_{23}e^{ikL}}{1-\rho^2 e^{2ikL}} \quad \text{-------- (2)}$$

where, $\tau_{12} = \frac{2(\frac{d}{\Lambda})}{(1+Y\left(\frac{d}{\Lambda}\right)^2)}$ is transmission amplitude of the incident plane wave to couple with the fundamental mode inside holes, $\tau_{23} = \frac{2Y(\frac{d}{\Lambda})}{(1+Y(\frac{d}{\Lambda})^2)}$ is transmission amplitude of the wave guide mode, and $\rho = \frac{(Y(\frac{d}{\Lambda})^2-1)}{(Y\left(\frac{d}{\Lambda}\right)^2+1)}$ is reflection amplitude of fundamental mode inside the hole (see additional information in ref.[4]). In deep subwavelength region $|\rho| = 1$, and

$|\tau_{23}| \propto 2(\frac{d}{\Lambda})$ or $\Lambda \propto 2d$. Hence the periodicity value is required to be proportional to two times the hole size for extraordinary transmission.

Further if w is the wall thickness between the two holes, the periodicity can be written as the sum of the hole size and the wall thickness,

$$\Lambda = \left(\frac{\lambda}{n}\right) + w \quad \text{----- (3)}$$

Thus the optimal wall thickness as per above notation, can be shown to be

$$w = (2p - 1)(\frac{\lambda}{n}) \qquad \text{------ (4)}$$

Direct reception of wave field using Laser Doppler Vibrometer (LDV)

The Laser Doppler Vibrometer (LDV) used in our studies, can be used to capture local wave fields including evanescent waves from the surface of the sample. However, the evanescent waves arising from the wave-defect interaction, if captured directly in this manner, would be weak and not be sufficiently strong to resolve sub-wavelength defect features. The metamaterial lens serves to not only transmit the wave field to the image plane, but also to amplify the components including evanescent fields at target frequencies.

In order to demonstrate this, we performed a simple line scan experiment, where the LDV was used to pick up the wave field signals directly from the far-surface of the aluminium sample, after being scattered by the of notch of size λ/25. In another case, the metamaterial was placed in between the sample and the LDV pickup. The overall experimental set-up was similar to the ones described in the paper, as illustrated in Supplementary Figure 2(a) below. Supplementary Figure 2(b) presents results of the line scan for the cases of direct pickup using LDV (labeled as 'without MM') and pickup mediated by the metamaterial (labeled as 'with MM'). We observe that the while an indication of the presence of the notch appears in the result even with direct LDV pickup, the two edges of the crack cannot be resolved. However, when we use the metalens, the backscatter due to specular reflection and crack-edge diffraction become discernible; this is because scattering generally happens at higher frequencies, and this information is amplified by the metamaterial.

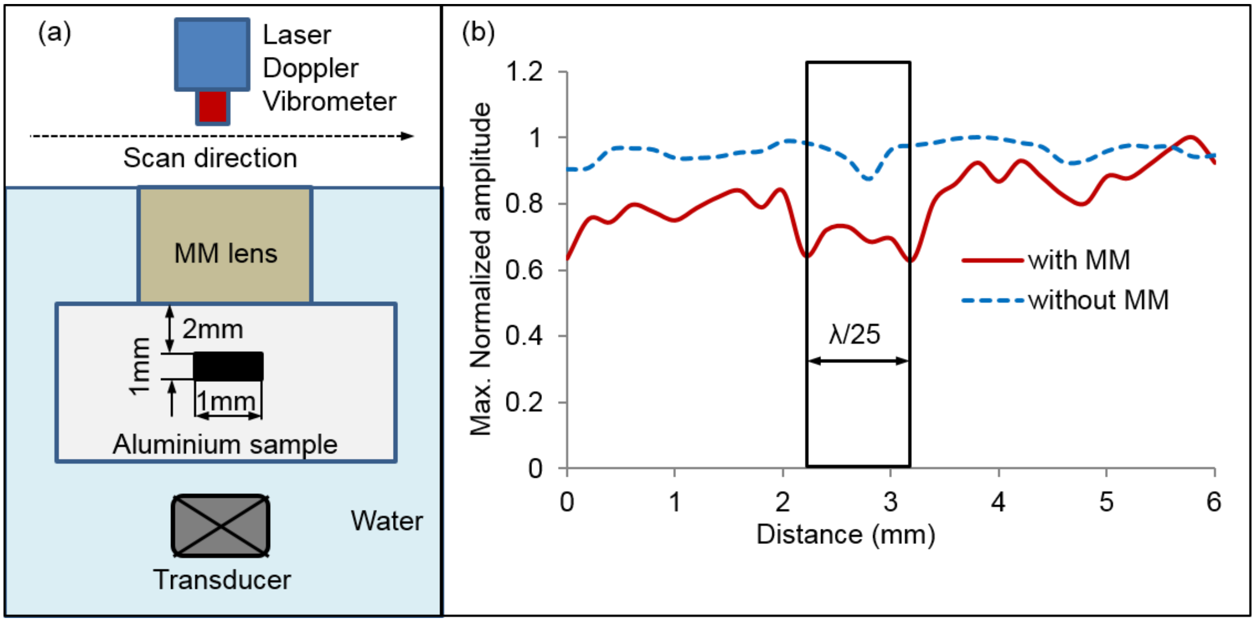

Supplementary Figure 2. (a) Illustration of experimental set-up (b) Experimental results for maximum normalized amplitude variation with monitored position across the sample, for direct pickup using LDV (labeled as 'without MM') and pickup mediated by the metamaterial (labeled as 'with MM'); the rectangular box indicates actual positions of the notch in the aluminium sample.

The above result can also be understood based on the physics of the interaction of evanescent waves with material boundaries/interfaces. Firstly we distinguish the following types of evanescent waves:

1. The first type consists of evanescent waves generated when homogenous (propagating) plane-crested waves impinge at a material interface or free boundary. In the context of bulk ultrasonic waves, several authors have studied this problem (see for example, Ref. [5])Evanescent waves generated due to such homogenous plane bulk elastic waves incident at interfaces are generally very weak and can be detected only at specific angles of incidence, e.g., close to Rayleigh critical angle.
2. The second type consists of the evanescent waves generated when plane-crested waves impinge at a defect due to the wave-defect interaction; the high-frequency evanescent waves generated due to wave-defect interaction that we are seeking to capture in our experiments are of this type.
3. Several authors have studied the interaction of evanescent waves with material boundaries, especially solid-fluid interfaces occurring in our case. Dechamps [6] for

example, shows that waves transmitted into fluid due to evanescent waves incident from solid side are generally weak, except at large incidence angles.

With this background, we observe that in the wave scattering problem studied in our paper, plane longitudinal ultrasonic waves interact with defects in a metallic sample with the following physical phenomena occurring:

- The main body of the normally incident (zero angle to interface normal) homogenous propagative wave field does not interact with the defect, and directly impinges on the material interface/boundary – based on Ref. [5], we know that such waves do not generate evanescent wave fields unless incident at angles close to Rayleigh critical angle (not valid in our problem as we consider normally incident plane waves).
- A part of the normally incident homogenous wave field interacts with the subwavelength defect, generating evanescent waves in the forward-scattered 'shadow' region of the defect. These evanescent waves then travel from the defect location to the surface of the sample – and it is these that we are seeking to capture.

However as per [6], such evanescent waves transmitted across solid-fluid interfaces are in general weak unless we look at large angles of incidence. Since the defects considered in our problem are of subwavelength dimensions, they can be treated as point-scatterers, and hence the forward-scattered wave fields will mainly be incident at normal angles (zero angles) at the interface. Thus, such defect-generated evanescent wave fields are very difficult to pick-up by regular methods. It is in picking up these waves that the metamaterial lenses are useful.

Optimal geometrical parameters of the metalens

Geometrical parameters of the metalens optimal for high transmission ratio (TR) were studied using finite element (FE) simulations, as described in the main body of the paper (page 3). However, in view of a large number of trial cases studied, only a subset of the results showing the variation of TR with certain illustrative or key values for the parameters were presented in Figure 1 in order to improve the readability. Supplementary Figure 3(a), (b) and (c) below present the full set of results, comparing the variation of TR with different values for diameter, length and periodicity of the holes, respectively.

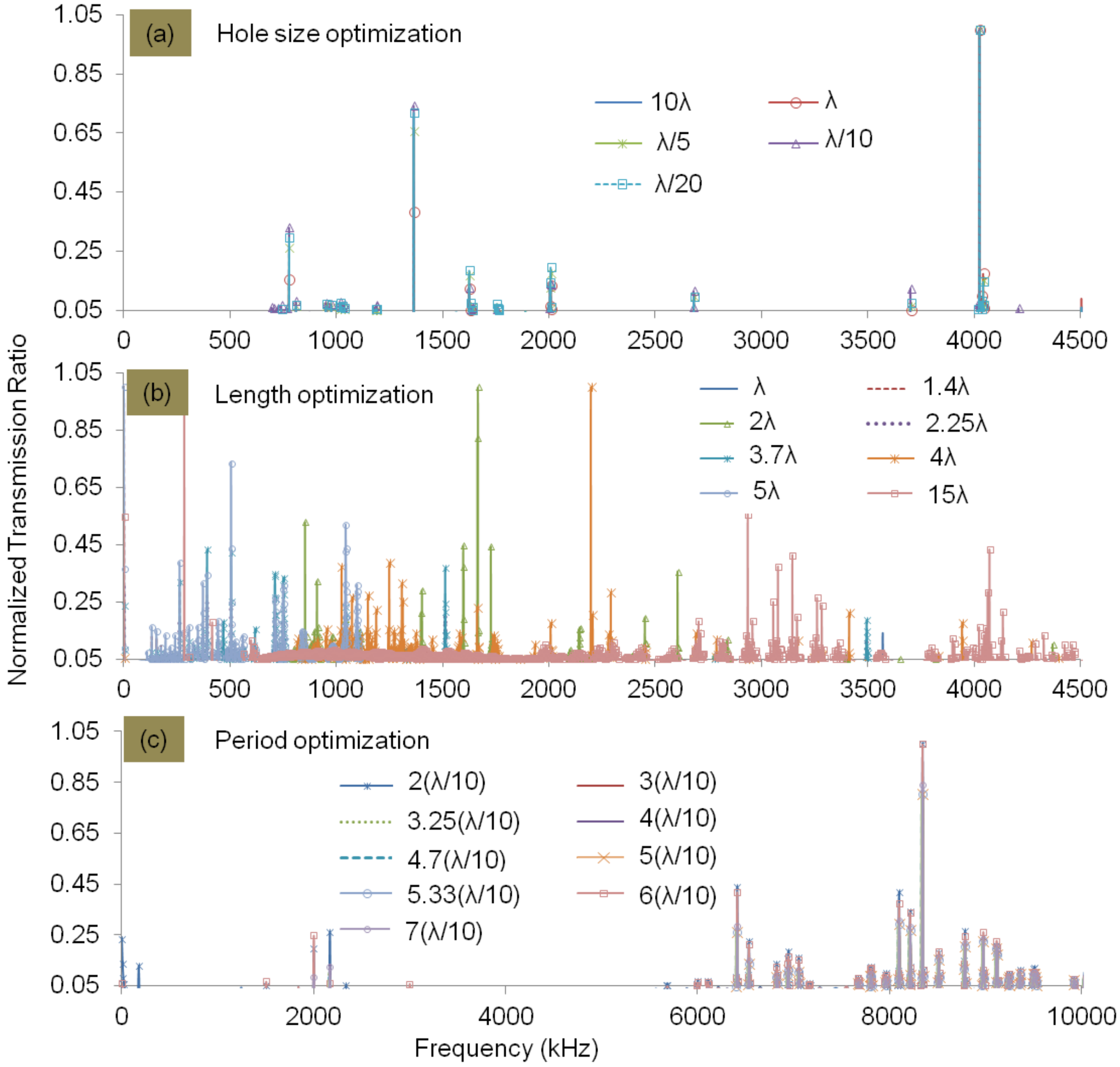

Supplementary Figure 3. FE results for Transmission Ratio (TR) of metamaterial with varying geometrical parameters of metamaterial lens, for different values of (a) Hole size (b) Hole length and (c) Periodicity (To avoid crowding of data points, for all above graphs the staring value on the ordinate is considered as 0.05 instead of 0). A subset of these results was presented in Figure 1 of the main body of the paper.

References:

[1] ABAQUS. Analysis User's Manual. Version 6.10-1, See website: http://www.3ds.com/products/simulia/portfolio/abaqus/abaqus-portfolio, accessed 28 July, 2015.

[2] Amireddy, K. K., Balasubramaniam, K. & Rajagopal, P. Holey-structured metamaterial lens for subwavelength resolution in ultrasonic characterization of metallic components. *Appl. Phys. Lett.* **108**, 224101 (2016).

[3] Sukhovich, A. *et al*. Experimental and theoretical evidence for subwavelength imaging in phononic crystals. *Phys. Rev. Lett.* **102**, 154301 (2009).

[4] Zhu, J. *et. al.* A holey-structured metamaterial for acoustic deep-subwavelength imaging. *Nature Physics* **7**, 52–55, (2011).

[5] Mott, G. Reflection and Refraction Coefficients at a Fluid-Solid Interface. *The Journal of the Acoustical Society of America* **50**, 819 (1971). [doi: 10.1121/1.1912706].

[6] Deschamps, D. Reflection and refraction of the evanescent plane wave on plane interfaces. *The Journal of the Acoustical Society of America* **96**, 2841 (1994). [doi: 10.1121/1.411290].
